# A structured framework for assessment of rehabilitation and sick-listing (STARS) – experiences among healthcare professionals

**DOI:** 10.1080/02813432.2026.2748807

**Published:** 2026-10-20

**Authors:** Eva-Marie Sundkvist, Inger Jansson, Annette Sverker, Elin Karlsson, Lena Rosenberg, Hanna Israelsson Larsen

**Affiliations:** a Department of Rehabilitation, School of Health and Welfare, Jönköping University, Jönköping, Sweden; b Department of Health, Medicine, and Caring Sciences, Linköping University, Linköping, Sweden; c Department of Activity and Health, Linköping University, Linköping, Sweden; d Pain and Rehabilitation Center, Linköping University, Linköping, Sweden

**Keywords:** Primary healthcare, rehabilitation process, rehabilitation coordinator, sick leave, sick-listing

## Abstract

**Background/purpose:**

The sick-listing process is often perceived uncertain and unclear by both patients and staff. To improve the process, a framework for structured assessment of rehabilitation and sick-listing (STARS) has been developed in the South-Eastern Healthcare Region in Sweden. STARS consists of seven methodological tools supporting healthcare professionals in sick-listing and rehabilitation. The purpose of this study was to investigate how primary healthcare staff clinically involved in the sick leave process experience working according to STARS.

**Method:**

We used a qualitative design conducting focus group discussions (*n* = 7) with healthcare staff (*n* = 37). Eligible participants were physicians, rehabilitation coordinators and other healthcare professionals working clinically with patients. The focus group conversations were analyzed with thematic content analysis according to Braun and Clarke. The study was registered in clinicaltrials.gov (number NCT05655962).

**Result:**

The participants’ experience of working according to STARS were described by the overarching theme ‘STARS as an enabling structure’ formed by three themes and eight subthemes. The themes were ‘Clearer roles and responsibilities through the RECO function’, ‘Moving from individual work to teamwork’ and ‘A more active and personalized rehabilitation and sick-listing process’.

**Conclusion:**

The findings suggest that implementing the STARS framework may provide a supportive structure for a uniform and consistent way of working across primary healthcare centers. By clarifying RECOs’ roles and strengthening interprofessional collaboration, the framework may support a more patient-centered approach across services.

## Introduction

In most welfare countries, paid sick leave serves to support recovery from illness [1–3] and protects individuals from income loss during work absence [3,4]. However, the sick leave system also entails considerable societal costs. In 2024, the Swedish Social Insurance Agency (SSIA) paid a total of 45,9 billion SEK in sick leave benefits, an increase of 12 billion SEK since 2019 [1]. The most common causes for sick leave are mental health problems and musculoskeletal disorders, consistently demonstrated across diverse populations both in Sweden and globally [1,5]. Sick leave due to these causes is also those most frequently prolonged, particularly in patients with complex or multifactorial health conditions [6]. Such extended sick leave periods are associated with several risks, including delayed return to work (RTW), future absence from work, unemployment, social exclusion, psychological problems and poor health outcomes [6–8]. The rising levels of sick leave, combined with a high risk of prolonged sick leave within major diagnostic groups, underscore the need for effective and well-coordinated rehabilitation and RTW processes to reduce both individual suffering and societal costs. To address these challenges, a STructured framework for Assessing Rehabilitation and Sick-listing (STARS) has been developed and integrated into primary healthcare in The South-Eastern Healthcare Region in Sweden.

In Sweden, the majority of medical certificates for sick leave are issued within primary healthcare, where patients initially seek care for symptoms and diseases that impair their ability to work. The sick leave and sick-listing process is regulated by law [9] and regulations [4,10,11] and involves multiple actors, both within and external to primary healthcare. Typically, the first point of contact for the patient is with a nurse answering the phone and booking an appointment with a physician who assesses how the patient’s condition affects their work capacity and issues the medical certificate [4,12]. Then, treatment and rehabilitation often involve several professionals beyond the physician, including nurses, occupational therapists, physiotherapists, psychologists, rehabilitation coordinators (RECO) and counselors depending on the patients’ needs [13,14]. The decision to grant or decline sick leave benefits is made by an SSIA official [4]. Although this multidisciplinary approach in Swedish primary healthcare enables comprehensive care, the whole process is often experienced as complex and challenges remain regarding continuity, role clarity and coordination for both patients and professionals [13,14]. In this manuscript, the term ‘sick leave’ describes the period of absence from work due to illness, while ‘sick-listing’ refers to the assessment of work capacity and issuance of the medical certificate.

Specifically, physicians often experience the sick-listing process as a complex and burdensome responsibility [15–20]. This responsibility is frequently carried out under time constraints and for patients with unclear or multifactorial health problems, requiring physicians to rely on non-medical competencies, such as knowledge of insurance medicine and occupational health [15,17,19,20]. The dual role of being both a treating physician and a gatekeeper to the social security system may cause loyalty conflicts and sick-listing has been described as a work environment problem [18]. Physicians have expressed a need for closer collaboration with other professions to clarify and improve the rehabilitation and sick-listing process [15,16]. Patients, in turn, often perceive the process as unclear, unstructured and characterized by uncertainty [8].

Since 2020, Swedish healthcare services are legally obligated to offer sick-listed patients rehabilitation coordination if needed [21]. The legislation is broadly formulated and lacks detailed guidelines, leaving room for local interpretations. Coordination is typically managed by a RECO, whose role includes monitoring sick-listing, rehabilitation and the RTW process. RECOs are usually professionals already employed at the healthcare center (eg occupational therapists, physiotherapists, nurses or social workers) who have received additional training and education in insurance medicine. The role is often part-time, combined with their primary profession at the healthcare center. Internationally, the results of introducing such a rehabilitation or RTW coordinator in the sick-listing process are diverse, ranging from no effect to strong positive outcomes on RTW, probably due to a wide disposition of different organizational settings and different working methods [22,23]. Thus, the RECO role remains ambiguous, both for the RECOs themselves and for other healthcare professionals, with insufficiently specified role descriptions contributing to uncertainty and variation in coordination practices [24–27]. Reports about the Swedish sick-listing and rehabilitation process has highlighted lack of clear routines contributing to stress and strain in clinical practice, identifying a gap between what is expressed in policies and guidelines and how the process is perceived to function in everyday practice [28–30].

This lack of clarity, together with the complexity of the sick-listing process, highlights the need for structured approaches that support coordination, define professional roles and promote efficient rehabilitation and RTW. The newly developed STARS framework is intended to meet these needs. The development and integration of STARS is described in detail in the Materials and Methods section. Understanding how key stakeholders, in this case healthcare professionals, interpret and apply such a new practice in their daily work has been identified as a crucial determinant for successful implementation in healthcare settings [31,32]. Hence, the study focuses on exploring how healthcare professionals experience working with STARS.

## Materials and methods

### Study design

This qualitative study had an exploratory design. Focus group discussions (FGD) were chosen to take advantage of the interaction between participants and to explore shared experiences and collective meanings that might not emerge in individual interviews, in line with methodological guidance for FGD [33]. The Consolidated Criteria for Reporting Qualitative Research (COREQ) checklist was used when planning, conducting and reporting the study [34].

### Research setting

This study was part of a larger pragmatic clinical trial in Swedish primary healthcare and took place in the South-Eastern Healthcare Region in Sweden which includes three regions, encompassing a total of 1.09 million inhabitants [35]. The nine primary healthcare centers, three in each region, that developed and implemented STARS, were all included in the study. The healthcare centers were located in areas with diverse socioeconomic conditions and varied in size. They served between 3500 and 11,700 listed patients (average 9600) and employed 14 to 91 staff members (average 81). The Care Need Index (CNI) ranged from 0.81 to 2.72 (average 1.46).

In Sweden, primary healthcare is provided through publicly funded healthcare centers. All centers employ general practitioners and nurses, while access to other professionals – such as psychologists, physiotherapists, counselors and occupational therapists – may be organized differently across centers. The assessment of work capacity during sick leave follows a three phased structure outlined in the Social Insurance Act [9], commonly referred to as ‘the rehabilitation chain’: up to 89 days, between 90 and 179 days and beyond 180 days [36]. In a European perspective, Swedish primary healthcare in general is characterized by a relatively low proportion of physicians and a wider range of healthcare professionals and a stronger emphasis on teamwork [14].

### The STARS framework – development and integration into practice

In spring 2020, the Regional Working Group on Insurance Medicine in South-east Sweden initiated the development of the STARS framework to support rehabilitation and sick-listing in primary healthcare. The first version was co-created with RECOs from the above mentioned nine pilot healthcare centers and researchers. Integration into practice began in autumn 2020, guided by the Swedish Public Health Agency’s implementation checklist [37]. Regional process managers coordinated planning with RECOs, local managers and physicians. Each center developed an integration plan using Tool 1 (Table 1) to map resources. RECOs acted as key facilitators, sharing information and supporting staff. Development and implementation ran in parallel, enabling iterative refinement. Healthcare professionals and researchers collaborated throughout, while process managers and RECOs held regular follow-ups. Three authors contributed to framework development but not to operational integration.

**Table 1.** Overview of the tools within the STARS framework.

Tool nr.	Applies	Content	Applied in rehabilitation chain, day:		
			1–89	90–179	180–
1.	Before implementing STARS.	Inventory of resources and needs at the unit. The inventory assesses the unit’s available professions, their roles in sick-listing and rehabilitation and routines for internal and external collaboration. It also examines guidelines, contact structures and whether these routines and forums are known and applied across the unit.			
2.	Day 1 in sick-listing	Establish a routine for regular checks of the unit’s patients that are sick-listed. Identify and contact patients in need of rehabilitation coordination.	x		
3.	Day 1–30 in sick-listing	Meeting with patients for a mapping interview either through phone or at a physical appointment at the healthcare center.	x		
4.	Day 31–89 in sick-listing	Establish and document a sick-listing and rehabilitation plan to enable RTW.	x		
5	When needed	Contact with external part – employer, SSIA, the Swedish Public Employment Service, or social services	x	x	x
6.	When needed	Multiparty meetings with patient and relevant internal and external actor (listed above).	x	x	x
7.	Day 90 and after in sick-listing	Follow-up meeting with patient and other relevant internal and external actors. Continued planning if needed.		x	x

STARS is a framework with seven tools supporting healthcare professionals in sick-listing and rehabilitation. Based on principles of high-level rehabilitation coordination [38], following a timeline aligned with the SSIA’s rehabilitation chain [36], it addresses patient support and collaboration with healthcare and external actors. STARS also responds to the gap between what is expressed in policies and guidelines and how the sick-listing and rehabilitation process is perceived to function in everyday clinical practice [28,29]. The framework is structured around the first 90 days of the sick-listing to minimize the risk of patients being overlooked in the rehabilitation process, while tools 5–7 also apply after 90 days. Tool 1 maps internal and external resources at the organizational level within the healthcare center and precedes tools 2–7, which are directed toward supporting individual patients. Tools 2–4 guide coordination during days 1–89 with a focus on identifying and meeting patients in need of coordination and establish a sick-listing and rehabilitation plan. Tools 5–6 focus on contacts with external actors such as employers and SSIA and can be applied throughout the entire sick-listing period, including beyond 90 days. Tool 7 covers follow-up and, if needed, continued sick-listing after 90 days. Each tool includes detailed checklists for tasks such as initial patient meetings, establishing routines for regularly reviewing the unit’s sick-listed patients and providing guidance on how to initiate contact with employers. The framework is adaptable to patient needs and local resources. An overview of the tools is shown in Table 1.

### Participants and recruitment

All healthcare professionals that were clinically involved in the rehabilitation and sick-listing process of patients at the nine included healthcare centers were eligible study participants. This included RECO, physicians, counselors, nurses, occupational therapists, physiotherapists and psychologists. Healthcare professionals who did not work clinically in the sick-listing process were excluded, eg receptionists and managers. To recruit participants, the managers of the healthcare centers were contacted by a research nurse who informed about the study, gave written information about the study and communicated the planned dates for the FGD. The managers then passed on the written information to all healthcare professionals meeting the inclusion criteria. Healthcare professionals interested in participating in the study then contacted the research nurse and in addition to the already provided written information they also received oral information before agreeing to participate. Then, invitations with a zoom link were sent out. In total, 39 individuals accepted the invitation. Following the cancelation of two participants, the final sample comprised 37 individuals.

### Data collection

Data were collected through seven FGD, involving participants from all nine healthcare centers working according to the STARS framework. Two FGDs were performed with RECO, two with physicians and three with mixed professions (see Table 2). The grouping of healthcare professionals was intentionally designed to foster open and in-depth dialogue among participants with similar professional backgrounds, while also minimizing potential power imbalances [39]. In total, seven focus groups with 3–8 participants per group from the nine healthcare centers were carried out. Five groups comprised participants from all three regions and two groups from two regions. The FGDs were conducted online and recorded with the zoom application. The FGDs lasted between 52 to 86 min (Table 2).

**Table 2.** Demographic characteristics of the participants in the focus groups.

Group	Number of participants	Number of regions represented	Number of healthcare centers represented	Gender	Professions	Minutes
1	6	3	5	2 Women, 4 men	Physicians	77
2	7	3	5	3 Women, 4 men	Physicians	86
3	5	3	5	5 Women	RECO	53
4	5	3	4	5 Women	RECO	56
5	3	2	2	3 Women	Mixed professions^a^	56
6	3	2	3	3 Women	Mixed professions^a^	56
7	8	3	5	7 Women, 1 man	Mixed professions^a^	66
Total	37			28 Women, 9 men		

^a^ Nurses, occupational therapists, physiotherapists, psychologists, social workers.

The first and second authors (EMS, IJ) were moderators in all groups. First, background questions were asked to all participants about profession, geographical location and number of working years in primary healthcare. The RECOs also received specific questions about working years as RECO and training/education in insurance medicine. Then, the FGD started with an open question asking the group to describe their experiences of working according to STARS. During the discussion the moderators asked probing follow-up questions to encourage informants in reflection and elaboration such as eg: ‘Can you give an example?’/’Can you describe that further?/Do you see any negative side effects?’. The moderators facilitated the discussion to ensure that each participant had the opportunity to contribute their views. One of the authors made short notes and, at the end of each FGD, summarized the discussion and the participants were then given the opportunity to add additional thoughts and reflections. The FGDs were transcribed verbatim by a professional transcriber. All information that could identify individuals or places was removed. Data was collected from November 2022 to April 2023.

### Researchers’ preunderstanding

All six authors were women from various professional disciplines (four occupational therapists, one physician, one social worker) with experience of working with sick-listing, rehabilitation and the RTW process within primary healthcare. However, none had experience from working as a RECO. Four of the authors were well familiar with the topic insurance medicine and three had been involved in the development of STARS. None had been involved in integration into practice of the STARS framework. All authors had previous experience from qualitative studies.

### Data analysis

To explore and understand the data, we conducted a reflexive thematic analysis following Braun and Clarke using an inductive, bottom-up approach [40]. This approach was chosen for its flexibility and suitable for identifying patterns across qualitative data, including FGD. Transcripts were read several times by the first and second authors (EMS, IJ) for familiarization. Initial semantic coding was jointly performed for the first five FGDs and individually by the first author for the last two. The authors then discussed codes for reflexivity. The first author organized codes and related text in a table, which was then merged and grouped into preliminary themes and subthemes. This iterative reflexive process was conducted collaboratively in a larger group (EMS, IJ, AS and HIL), involving repeated movement back and forth between the data, codes and emerging themes. The themes were then refined, defined and named by the first author and discussed with all authors to ensure multiple perspectives. The first author then revisited the dataset to confirm alignment with the data. Finally, all authors reviewed and finalized themes. An example of this process is shown in Table 3.

**Table 3.** Examples of data analysis from data extract to themes.

Data extract	Codes	Subthemes	Theme	Overarching theme
‘I don’t need to focus on everything; I can hand over certain things to her [the RECO]. I can focus much more on the medical aspects and not the socioeconomic part’. (FGD 2)	Focus on treatment rather than insurance medicine	Enabling staff to focus on their core commission.	Clearer roles and responsibilities through the RECO function	STARS as an enabling structure
‘The RECO identify cases, and then they act quickly – like, ‘we might need to involve a physiotherapist here’, or ‘we need a psychologist’, or ‘we need to think in a multiprofessional way right from the start’. (FGD 5)	RECO initiates teamwork from the beginning	Teamwork that facilitates a multifaceted picture	Moving from individual work to teamwork	
‘We [the RECO] can be involved in helping and supporting the planning process, and also get the patient engaged in their own planning - and that usually tends to be quite positive’. (FGD 4)	Engaging the patient in the rehabilitation planning	A more informed and engaged patient	A more active and personalized rehabilitation and sick-listing process	

## Results

An overarching theme titled ‘STARS as an enabling structure’, was constructed to capture how the STARS framework – sustained by the RECO – facilitated team collaboration and had impact on the patients’ rehabilitation process. Across all focus groups, participants expressed strong consensus and a positive attitude toward working according to the STARS framework; despite repeated prompts from moderators to explore potential drawbacks, few negative consequences were identified. Questions about challenges or negative experiences of STARS primarily gave examples that were either unrelated to the framework itself, concerned earlier experiences before the introduction of STARS or referred to practices originating outside the participants’ own units. The discussions were often characterized by nods of agreement and participants building on each other’s experiences, reinforcing a collective understanding of the STARS framework. The overarching theme encompasses three themes; ‘Clearer roles and responsibilities through the RECO function’, ‘Moving from individual work to teamwork’ and ‘A more active and personalized rehabilitation and sick-listing process’. Together, these themes, supported by eight subthemes, illustrate how the participants experience the STARS framework as a practical and flexible tool that enhances coordination, teamwork and patient-centered care. The relationship between the overarching theme, the three themes and their subthemes is illustrated in Figure 1. When a topic was discussed in all focus groups, it is referred to as ‘all focus groups’ or ‘all participants’. When discussed by specific groups, they are referred to as ‘physicians’, ‘RECOs’ and/or ‘mixed healthcare professions’.

**Figure 1.**
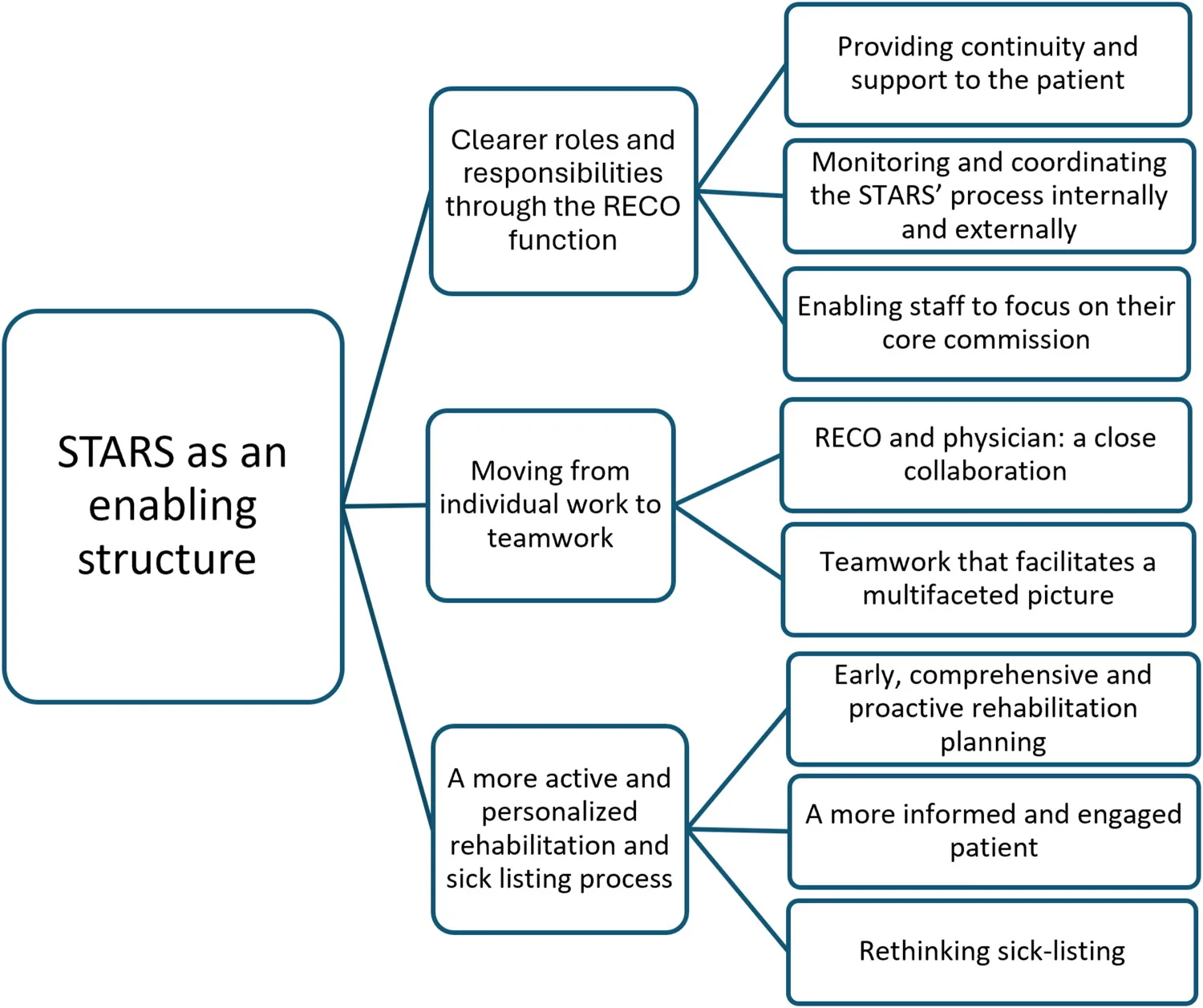
Overarching theme, themes and subthemes.

### Theme 1: clearer roles and responsibilities through the RECO function

All focus groups emphasized how the implementation of STARS clarified responsibilities in the rehabilitation and sick-listing process. The RECO was described as a central actor in coordinating, monitoring and supporting the process enabling other healthcare professionals to focus on their own area of expertise

I think the structure has become clearer for everyone. Our RECO probably had a structure before as well, but the rest of us may not really have known about it. (FGD 6)

Although RECOs sometimes experienced stress due to a high workload, they also expressed feeling appreciated. They felt positively about the high level of trust placed in their role by physicians and other healthcare professionals and noted that their expertise was in high demand. Physicians, in turn, acknowledged the demanding nature of the RECO role and reflected on how the workload might affect RECOs’ well-being.

### Providing continuity and support to patients

All focus groups described the RECO as more easily accessible to patients than, for example, physicians. Patients were often given a direct telephone number to the RECO, which according to the participants’ experiences was highly valued. The RECO was described as a linking element between patients and physicians. In healthcare centers with few permanent physicians, the RECO role was perceived as contributing to continuity of treatment and rehabilitation.

RECOs described how the STARS framework clarified and structured their close work with patients enabling a more standardized approach across the different healthcare centers. They described having time to motivate patients, to explain the purpose of their sick leave and to offer support and follow-up throughout the gradual RTW-process.

There can be a lot of anxiety before the first step-up [in returning to work] - we [RECOs) can call the patient before start, to have that final motivational conversation beforehand. But we also have the possibility to call after they’ve been back for a while, to check in and see how it has gone. (FGD 4)

### Monitoring and coordinating the STARS’ process internally and externally

The focus groups described the RECO as playing a leading role in monitoring the STARS’ various steps and functions. The RECOs were seen as a control mechanism, overseeing sick-listing cases and ensuring that patients in need of internal or external interventions were not overlooked.

RECOs noted that the STARS framework provided a clearer structure for their collaboration with external actors such as employers and SSIA as well as for the work at the organizational level, including monitoring teamwork at the healthcare centers.

We [RECO] become like a control function, where we can identify patients who had received sick-listing without a proper plan, for example from a temporary physician. Then we can step in and guide the process – so it doesn’t escalate out of control.

Yes, exactly

Precisely…. (FGD 4)

The RECO’s expertise in insurance medicine and the sick-listing process was recognized as key sources of knowledge within the team and in patient interactions. They often introduced new staff and temporary physicians to the healthcare center’s approach to sick-listing. Their broad understanding of various healthcare professions enabled them to match patients with appropriate professionals early, avoiding passive sick leave periods without rehabilitation planning. RECOs were also central in the external collaboration, such as with SSIA and employers.

### Enabling staff to focus on their core mission

Physicians and mixed healthcare professionals described the sick-listing process as time-consuming. However, the latter group reported lacking sufficient knowledge of rules and regulations governing insurance medicine. Both groups expressed that the implementation of STARS, along with the RECO function contributed to clearer professional roles and responsibilities within the rehabilitation and sick-listing process. This, enabled them to focus more on clinical work, while delegating parts of the sick-listing process to the RECO. They appreciated being relieved of administrative burdens and noted that they were less frequently required to handle questions from and attend meetings with employers or officials from the SSIA. This clearer allocation of responsibilities allowed them to dedicate more time to patient treatment and rehabilitation, thereby improving the quality.

I find it quite relieving that it allows you to focus on the treatment rather than on issues related to insurance and work. This statement led to nods in the group, indicating a consensus. (FGD 6)

I don’t need to focus on everything; I can hand over certain things to her [RECO] and concentrate more on the medical aspects. (FGD 2)

### Theme 2: moving from individual work to teamwork

In the focus groups it was discussed that they had experienced a shift from individual work to more collaborative approaches, which they appreciated. They emphasized that teamwork is a fundamental component of the STARS framework.

### RECO and physician: a close collaboration

Participants highlighted the intensified collaboration between physicians and RECO, noting that they worked more closely together both through scheduled recurring meetings and informal ad hoc meetings. During these meetings, they reviewed the physician’s register of sick-listed patients to ensure that each patient had an appropriate and updated plan. Physicians found these briefings helpful, as they provided insight into the patient’s situation and increased their general awareness of the rules and regulations governing the sick-listing process.

The initial mapping interview conducted by the RECO (tool nr 3), prior to physician’s first appointment with the patient was highly appreciated. All participants described these interviews as having a broader focus, including aspects such as work capacity and socioeconomic factors such as employment, family situation and social support. Physicians experienced this background information useful for planning treatment and issuing the sick-listing certificates, as it facilitated more efficient consultations.

The contact with the patient, the patient’s connection with healthcare, and how everything just keeps improving - and I’ve learned so much from the RECO about the system, how it all works, what possibilities exist, and where to turn to. They are a huge support in all of this. I don’t want to work without them, so I really hope they’ll always be around - and that this way of working remains, because it suits us so well. (FGD 2)

### Teamwork that facilitates a multifaceted picture

The growing interprofessional collaboration was described as an essential part of STARS. Some healthcare centers held regular team meetings, while others organized them when needed. The meetings served various purposes, from direct planning with patients to organizational-level coordination, such as reviewing sick-listing cases and identifying patients suitable for multiprofessional assessment. Such meetings were perceived as reducing the risk of patients being overlooked and as important forums for sharing information, including updates on insurance medicine regulations, discussing complex cases and planning further interventions. They also supported interprofessional learning and were helpful when introducing new healthcare professionals to the workplace.

Participants emphasized that teamwork strengthened continuity and quality through improved communication regarding rehabilitation and sick-listing within the primary healthcare center. A shared understanding of routines and working methods was described as crucial for high-quality work and should be established prior to implementing STARS, facilitated by management. All focus groups highlighted that involving multiple healthcare professionals in assessment, planning, treatment, rehabilitation and decision-making improved the quality of both rehabilitation and sick-listing. Physicians valued multiprofessional assessments for providing a more comprehensive understanding of patients, especially in complex cases and reported feeling more supported in their work.

I feel that we’re quite united at the healthcare center in thinking that it’s helpful to do this together – that we reinforce each other’s messages. For example, I might start preparing the patient for returning to work during my appointment, which then makes it easier for the physician to follow up on. And vice versa – the physician might suggest it’s time to go back to work, to avoid falling into avoidance behavior – and that makes it easier for me to work with it in psychological treatment later on. (FGD 6)

### Theme 3: a more active and personalized rehabilitation and sick-listing process

Participants across all focus groups reported that the STARS framework placed greater emphasis on rehabilitation and sick-listing within the primary healthcare center. Physicians perceived that sick-listing had become easier to manage and more patient-safe, as the structured approach ensured similar assessments across cases. Participants described how patients were now involved and seemed empowered, with a plan for the sick leave period established from the start.

### Early, comprehensive and proactive rehabilitation planning

Focus group discussions emphasized that compared to before STARS, rehabilitation planning started earlier and was more proactive, outlining steps for rehabilitation and gradual RTW. The RECO was usually involved from the beginning, although some RECOs felt they were sometimes included too late in complex cases or when difficult decisions about sick leave had to be communicated. To enable this early planning, participants discussed the importance of a comprehensive assessment of the patient’s situation. Rehabilitation interventions also began earlier, which the participants recognized initially could feel stressful for patients. However, they emphasized that STARS’ structured approach with clear planning, active patient involvement, explaining the purpose of sick leave and maintaining flexibility helped reduce this stress.

We start planning from day one, even if that planning may feel stressful and hard for the patient to accept. If we all – across all professions, the RECO, myself, and the physician – share the same perspective, support the patient, follow them closely, and maintain that contact, then the rehabilitation becomes more successful and faster.

Yes, the earlier you step in and make a clear plan, the easier it becomes….that’s really important. (FGD 7)

RECOs reported increased ambition to follow the framework and do the right things at the right time. They discussed that STARS is an overarching framework that needs to be individually adapted. Continuous patient contact, from early engagement through 90-day follow-ups and beyond, was described as demanding, but essential for quality.

### A more informed and engaged patient

Participants across all focus groups described that working according to the STARS’ framework was, in their view, beneficial for the patients. They outlined routines for informing patients about the RECO role, typically during the patient’s first contact with the healthcare center. These routines varied: in some healthcare centers, physicians provided the information, while in others, RECO sent written materials. The patients could also be directly directed to the RECO when calling the healthcare center and speaking with a nurse about sick leave.

Compared to previous experiences, they noted that patients were now more involved from the beginning, which they believed led to better preparedness for rehabilitation. Patients seemed empowered to take responsibility for their rehabilitation, receiving support when needed, for example in communication with employers. For patients with mental health problems or complex needs, participants emphasized that a structured and predictable process was crucial to create clarity and safety.

But when it comes to stress-related diagnoses, or depressions, it’s very important to provide a sense of safety, certainty, and control – and that’s exactly what the patient gets, when there is a clear plan in place from day one. (FGD 7)

### Rethinking sick-listing

Physicians and mixed healthcare professionals reported increased awareness and knowledge following STARS implementation. Although some participants in the mixed group were unfamiliar with the specific term ‘STARS’, they described working in a structured way with sick-listing, characterized by early rehabilitation planning, interventions, teamwork and collaborating with RECO, which they felt reflected the framework’s principles.

Both the STARS framework and the RECO were perceived as helpful in navigating the sick-listing process. Sick-listing was no longer seen as a single solution, but rather as one option alongside treatment and rehabilitative efforts. According to participants, the duration of sick leave had become shorter, with more patients on part-time sick leave compared to before STARS. Fewer patients received sick-listing as a quick fix without proper planning. Direct referrals to other healthcare professionals, such as physiotherapists or psychologists, enabled earlier rehabilitation and sometimes avoided sick-listing altogether.

The nurses triage the patients to the psychosocial unit when appropriate, and to the physiotherapist or occupational therapist when appropriate, which means that the patients don’t primarily come into contact with us physicians who have the authority to prescribe and issue a sick certificate. (FGD 1)

Participants discussed that the goal was not to eliminate sick-listing entirely. Temporary sick leave was still seen as necessary for recovery. What had changed, according to participants, was that rehabilitative and supportive interventions, such as occupational therapy, physiotherapy and psychological treatment, were initiated earlier and in a more structured and purposeful manner, often alongside or as an alternative to sick-listing.

## Discussion

This study explored how healthcare professionals at nine primary healthcare centers within the South- Eastern Healthcare Region in Sweden experienced working according to STARS. The results showed that STARS was perceived as a supporting structure closely linked to the RECO function, that facilitated continuity, collaboration and patient-centered treatment and rehabilitation. It enabled professionals to adopt a more active and individualized approach to rehabilitation and sick-listing, with the purpose of empowering patients to take an engaged role while allowing professionals to adjust the process based on individual needs. However, whether the patients felt empowered or not needs to be investigated in another study. Two foundational pillars of STARS – the RECO and the collaborative teamwork – was perceived to contribute to both strengthening interprofessional collaboration within the healthcare centers and improving coordination with external actors, such as the SSIA, by enabling early planning, ensuring more structured and multiprofessional assessments and reducing the risk of passive or prolonged sick leave through early intervention and structured follow-up. The consistent positive views across the groups may suggest that the STARS framework met needs that participants experienced in their daily work. Additionally, some healthcare professionals’ core expertise may be more closely aligned with return-to-work processes than that of physicians, which could explain the overall positive attitude. RECOs, who often have a more rehabilitation-oriented professional background, may perceive their responsibilities as well aligned with their core expertise.

## Structured coordination and teamwork through STARS

The STARS framework with suggestion of tools through the sick-listing process (Table 1) was perceived as providing a clear structure for coordination and teamwork in the sick-listing process. Physicians and other healthcare professionals expressed relief, noting that this allowed them to focus more on clinical treatment and rehabilitation. From a professional boundary’s perspective, the clarification of the RECO role through STARS may have contributed to greater clarity regarding how medical insurance responsibilities are organized and shared in primary healthcare, responding to the challenges related to the sick-listing process [18]. This structured form av coordination may enhance efficiency and patient safety, aligning with previous research portraying the RECO as ‘a savior’ [27,p.4] and ‘the missing piece of the puzzle’ [41,p.4]. Although the RECO role existed prior to STARS, participants emphasized the framework helped systematize and make RECO tasks more visible in everyday practice. This reflects a broader need for role clarity and optimal use of professional competence in the sick-listing process, as highlighted in earlier studies [15,16,19,24,42]. The STARS framework may address these needs by providing a formal structure for coordination that supports the RECO role and promotes teamwork and shared responsibility. By offering explicit examples of rehabilitative interventions, the framework may also serve as a tool that empowers RECOs to initiate actions involving other professionals. The framework may thus bridge the gap between vague, general guidelines and concrete application in real world settings [30].

However, the redistribution of tasks may lead to an increased workload for the RECO. While RECOs in this study expressed appreciation for the trust placed in their role, they also acknowledged its demanding nature. Previous research has linked unclear role descriptions and lack of coordination strategies to stress and role ambiguity [24–27]. In this regard, even though STARS appears to help reduce some of these challenges by clarifying expectations and work processes, the long-term sustainability of the RECO role requires continued attention to workload, support structures and organizational clarity.

Interprofessional collaboration in healthcare aims to improve care quality and optimize resource use [43,44]. Although it is a cornerstone of Swedish primary healthcare, challenges remain regarding continuity, role clarity and coordination [13]. In this context, STARS was perceived as providing a shared foundation for collaboration, enabling more coherent and structured teamwork. Its interprofessional approach was considered valuable for decision-making and comprehensive patient care, especially in assessing work capacity among sick-listed patients – a complex task that benefits from input from occupational therapy, physiotherapy and psychosocial expertise [19]. These results, aligned with previous research, show that team-based sick leave management can improve patient outcomes and help physicians better capture the multifaceted nature of work capacity assessments [24,42]. Shared routines and interprofessional learning appear central to effective teamwork in primary healthcare, consistent with studies emphasizing managerial support and clear structures [45,46]. Overall, the results of our study suggest that the STARS framework aligns with national and international recommendations highlighting coordination and interprofessional collaboration as key to improving care quality and efficiency [28,29,47,48].

## STARS as a bridge between healthcare and social insurance legislation

In addition to strengthening internal collaboration within primary healthcare, the STARS framework can also be understood as a practical tool that aligns with the legislative structure governing sick leave and sick-listing in Sweden, particularly the three-phased model defined in the Social Insurance Act (‘the rehabilitation chain’) [9]. The framework appears to facilitate the collaboration between healthcare providers and external actors such as SSIA by clarifying roles, responsibilities and expectations. In Swedish primary healthcare, according to the Act on Coordination Efforts for Patients on Sick Leave [21], healthcare providers are responsible for offering coordination support when needed to facilitate RTW. In contrast, the Social Insurance Act assigns SSIA a strategic role: to oversee and ensure that rehabilitation efforts are coherent and timely between stakeholders, including employers, healthcare and social services [9]. This distinction between responsibilities is central: healthcare carries the operational responsibility for coordination, while SSIA holds the supervisory role from a social insurance perspective. However, recent evaluations suggest that SSIA struggle to fulfill its responsibilities due to limited authority and insufficient collaboration with employers and healthcare providers [49]. In this context, the STARS framework may help address some of these shortcomings, both by offering a structured process within healthcare that supports healthcare as well as social insurance legal mandates and also effectively serve as an interface between clinical practice and administrative requirements, enabling more systematic interaction with SSIA and other external actors. Thus, STARS may help balance the legal and regulatory requirements of the social insurance system with patient-centered care by providing a structured yet flexible approach that supports early return to work and individualized rehabilitation planning, as outlined in Table 1. The close alignment between social insurance timeframes and the STARS structure may reduce the risk of patients being overlooked in the sick-listing process. At the same time, an overly strong adherence to medical insurance regulations within primary healthcare may risk shifting focus away from care and rehabilitation needs. However, in the STARS framework suggestions for rehabilitative efforts are an integrated part with the medical insurance requirements, which may support earlier identification of rehabilitation needs. Nevertheless, there remains a potential risk that rehabilitation and treatment processes become too closely aligned with insurance regulations, potentially at the expense of individualized care. While early RTW is often advocated in both policy and practice, evidence for the principle of ‘the faster the better’ remains mixed. It is not only the timing of RTW that matters, but also the timing of rehabilitative interventions in primary healthcare [50]. Swedish RTW research points to the challenges of identifying clear effects of early coordinated interventions in primary healthcare, indicating that timing alone may be insufficient to account for RTW outcomes [51,52]. Also, a quasi-experimental study showed that longer rehabilitation and longer RTW-process resulted in more sustainable employment than shorter rehabilitation and faster return to work [53]. These two aspects, early RTW and early rehabilitation, require further investigation, particularly in relation to how STARS can balance these priorities without compromising patient-centered care.

## Methodological considerations

To ensure trustworthiness, this study was informed by principles of qualitative rigor and reflexivity, drawing on Tracy’s ‘Big Tent’ framework as a guiding lens [54].

FGDs were well suited for exploring how healthcare professionals experienced the STARS framework. Interaction occurred in all groups, and despite variations in size and the use of Zoom, discussions were dynamic and generated rich material. Smaller groups allowed deeper engagement, while larger groups provided broader perspectives. Although online formats can limit spontaneity [55], the practical benefits of enabling participation across geographically spread centers outweighed these drawbacks.

Professionals from all nine healthcare centers, across relevant professions were invited and 37 participated in an FGD, providing diverse perspectives that strengthen the credibility of the results. Participation was self-selected and while we have no reason to assume major differences in experiences among those who declined, there is still a risk of sampling bias and that the result does not represent everyone’s experience of working according to STARS. Participants taking part were purposefully grouped by professions to support open dialogue and reduce potential power imbalances. This approach appeared effective, as moderators noted that discussions were generally open and respectful, with little indication of hierarchical influence.

Three of the authors had prior involvement in developing STARS, which provided contextual insight but also posed a risk of bias. This was addressed through an ongoing reflexive dialogue and by also including authors without prior involvement, supporting confirmability through reflexive awareness of pre-understanding. Another potential source of bias concerned social desirability bias, which may be especially relevant in an implementation context. All the RECOs who participated in the focus group discussions have also been involved in the development of the STARS framework. This may have influenced their responses to be more positive. However, their discussion took place in profession-specific groups, which likely reduced the risk of wanting to defend STARS in front of colleagues. A similarly positive tone was also observed in the physician group and among participants from other professional backgrounds. To reduce the risk of social desirability bias, the moderators consistently included follow-up questions addressing challenges and negative experiences with STARS across all FGDs. Nevertheless, group dynamics, along with the presence of researchers as moderators, may have contributed to reinforcing shared positive interpretations.

To support dependability and methodological rigor, the research process is transparently described, and the COREQ checklist was used to guide transparent and comprehensive reporting [32].

Results are most likely transferable to all Swedish primary healthcare settings, since the sick-listing process is similar across the country. The results may also be transferable to other Western healthcare systems with similar rehabilitation and sick-listing structures. Ultimately, transferability must be assessed by the reader.

## Implications for practice

The results suggest that STARS can help integrate sick-listing within the broader rehabilitation process, enhance coordination, patient involvement and clinical decision-making in primary healthcare. A key strength is its ability to promote clarity of roles, establish shared routines, strengthen interprofessional collaboration and better use of professional competence across the team, potentially improving continuity of patient care. The STARS framework appears to function not only as a structural model but also as a conceptual and reflective tool that fosters awareness, critical thinking and a more systematic approach to sick-listing and rehabilitation. By linking sick-listing more directly with rehabilitative interventions, a more cohesive and person-centered approach to care might be achieved. When teamwork is improved considering one patient group such as the sick-listed patients in STARS, it could also lead to synergistic effects and enhance collaboration at the healthcare center as a whole. However, further research is needed to explore whether and how these potential spillover effects influence teamwork and care quality more broadly in primary healthcare settings, such as other patient groups not currently sick listed.

## Future research directions

Perceived reduction in sick leave duration and extent suggests that the structured approach in STARS may influence sick leave patterns. However, further research is required to investigate the long-term effects of STARS on patient outcomes and healthcare resource utilization. Specifically, quantitative studies should assess whether the perceived improvements in care quality, coordination and team collaboration can be translated into measurable outcomes, such as RTW-rates, reduced recurrence of sick leaves, or more efficient use of healthcare services. Patients’ experiences and satisfaction with the rehabilitation and sick-listing process should also be investigated to assess whether the structured approach supports person-centered rehabilitation. In addition, the role of the RECO warrants closer examination, particularly in relation to workload, role clarity and its impact on stress and job satisfaction. The results also highlight the need for a deeper understanding of physicians’ work environment in the context of sick-listing. Given that sick-listing is often experienced as a complex and burdensome task, future studies should explore how STARS influences physicians’ perceptions of their clinical responsibilities, stress levels and overall work satisfaction.

## Conclusions

The results indicate that the STARS framework was experienced as a supportive structure. It promoted uniform and consistent work across primary healthcare centers. For instance, by providing increased role clarity among RECOs, strengthening interprofessional collaboration and promoting a more patient-centered approach. These results suggest that STARS has the potential to improve quality of the rehabilitation and sick-listing process as well as strengthen the link between healthcare and the social insurance system. Further research is needed to assess its long-term impact on patient outcomes, return to work-rates and staff work environment.
